# Augmented reality visualization for ultrasound-guided interventions: a pilot randomized crossover trial to assess trainee performance and cognitive load

**DOI:** 10.1186/s12909-024-05998-8

**Published:** 2024-09-27

**Authors:** Shu-Chen Liao, Shih-Chieh Shao, Shi-Ying Gao, Edward Chia-Cheng Lai

**Affiliations:** 1 Department of Emergency Medicine, Keelung Chang Gung Memorial Hospital, Keelung, Taiwan; 2 Chang Gung University College of Medicine, Taoyuan, Taiwan; 3 Department of Emergency Medicine, Linkou Chang Gung Memorial Hospital, Taoyuan, Taiwan; 4 Department of Pharmacy, Keelung Chang Gung Memorial Hospital, Keelung, Taiwan; 5 School of Pharmacy, Institute of Clinical Pharmacy and Pharmaceutical Sciences, College of Medicine, National Cheng Kung University, Taiwan, No.1, University Road, Tainan City 701, Tainan, Taiwan; 6 Population Health Data Center, National Cheng Kung University, Tainan, Taiwan

**Keywords:** Augmented reality, Ultrasound-guided central venous catheter (CVC), Point-of-care ultrasound (POCUS), Cognitive load, NASA Task Load Index, Medical education

## Abstract

**Background:**

Augmented reality (AR) technology involving head-mounted displays (HMD) represents a significant innovation in medical education, particularly for training in guided invasive procedures. Novice physicians often face challenges in simultaneously identifying anatomical landmarks and performing procedures when learning point-of-care ultrasound (POCUS). Our primary objective was to determine the effectiveness of AR in enhancing physician training for ultrasound-guided interventions using AR visual overlays. The secondary objective was to compare cognitive load between traditional ultrasound training settings and AR-assisted training settings.

**Methods:**

This randomized crossover study, conducted from 2021 to 2022, compared performance and cognitive load of trainees attempting accurate central venous catheter (CVC) placement using an AR-HMD to display ultrasound images (AR-US), compared with standard ultrasound without visual overlay (S-US). We enrolled 47 trainees, including 22 final-year undergraduate medical students and 25 postgraduate physicians (years 1–4) from three hospitals in Taiwan. All participants had basic training in US-guided CVC placement but lacked AR-US experience. Performance and cognitive load were assessed using time measurements and NASA Task Load Index (NASA-TLX), covering the dimensions of Mental-, Physical- and Temporal Demand, and Performance, Effort and Frustration.

**Results:**

We found AR technology reduced the time required for critical steps in CVC placement, while minimizing users’ neck strain. Female and junior trainees using AR-US identified anatomy and localized veins faster than those using S-US. Using AR-US, female trainees significantly outperformed males in anatomical identification [mean difference (MD): -10.79 s (95% CI: -2.37 to -19.21)]. The NASA-TLX cognitive load assessment showed mental workload trending lower in all dimensions except performance while using AR-US, compared to S-US. Similarly, junior trainees’ effort- and frustration scores were lower [MD: -2.73 (95% CI: -5.04 to -0.41) and -2.41 (95% CI: -4.51 to -0.31), respectively], as were female trainees’ effort scores [MD: -3.07 (95% CI: -6.10 to -0.03)] when using AR-US, compared to S-US, whereby these differences were statistically significant.

**Conclusions:**

AR technology helped improve trainee performance and reduced cognitive load during ultrasound-guided CVC placement. These findings support the application of AR technology to enhance physician training in ultrasound-guided interventional procedures, suggesting that AR-HMDs could be a valuable tool in medical education.

**Trial registration:**

The trial was registered with Clinicaltrials.gov on 20/09/2023 as a clinical trial, under the identifier NCT 06055400.

**Supplementary Information:**

The online version contains supplementary material available at 10.1186/s12909-024-05998-8.

## Background

Augmented reality (AR) technology is increasingly being adopted in the medical field, especially for training and clinical practice such as surgery and medical imaging [1–5]. A significant advantage of AR lies in its ability to create hybrid learning environments that combine both physical and digital objects, resulting in an immersive learning experience [6]. Recent studies have shown the advantages of AR head-mounted displays (AR-HMDs) for puncture guidance [3, 7, 8]. Al-Abcha A et al. utilized a monocular HMD in the catheterization laboratory for invasive coronary angiography or right heart catheterization. This approach involved puncturing the right radial artery and the right internal jugular vein, allowing the augmented reality system to seamlessly integrate both ultrasound and physical visual cues into a unified field of view [9]. A study conducted in South Korea on pediatric anesthesia surgery indicated that senior pediatric anesthesiologists using AR smart glasses (AR-HMD) for radial arterial catheterization outperformed their counterparts using conventional ultrasound [10]. In a follow-up study conducted in 2024, the same group of researchers examined the impact of trainee seniority on the effectiveness of AR smart glasses in a controlled experimental setting. The findings revealed that the use of AR smart glasses significantly enhanced the first-attempt success rate among junior anesthesiology trainees, decreased the number of attempts required, and reduced overall complication rates in pediatric patients [11]. AR-HMDs can benefit both educational and clinical settings by enhancing eye-hand coordination, providing real-time visual aids and reducing cognitive load [12].

Learning involves transferring information from sensory memory to working memory and, eventually, to long-term memory. Working memory acts as a filter, allowing only relevant information to be transferred to long-term memory, but its capacity is limited. When cognitive load exceeds working memory capacity, it can negatively impact learning and performance [13]. Cognitive load theory is based on the human memory model that includes three subsystems: sensory, working and long-term memory. Studies indicate that by modifying the split-attention effect [14, 15], the worked-example effect and guidance-fading effect, cognitive load can be reduced and learning enhanced [16, 17]. The split-attention effect posits that learning and performance can be enhanced by presenting information in an integrated format. In physics education studies, researchers utilized AR technology to convert traditional displays into virtual representations anchored to corresponding objects within the experimental setup, thereby producing an integrated AR view of real-time data. This AR condition significantly reduced extraneous cognitive load by mitigating the split-attention effect, compared to the traditional condition [18, 19]. AR provides a technology to achieve such integration, potentially reducing cognitive load and enhancing learning outcomes [16, 20, 21].

Ultrasound (US) guidance is commonly utilized in medicine for various procedures, including but not limited to injections, biopsies and cannulations. US-guided vascular access in point-of-care ultrasound (POCUS) improves success rates, reduces complications and decreases procedure time. However, its effective implementation often requires considerable expertise and experience [22, 23]. Studies have demonstrated that elevated cognitive load during POCUS simulation training is associated with diminished learning outcomes and inadequate transfer of skills to clinical practice [24]. Consequently, assessing cognitive load can be instrumental in identifying individuals whose competencies have not been thoroughly consolidated [14]. US-guided central venous catheter (CVC) placement is complex and may exceed physicians’ working memory capacity. In US-guided CVC placement, the first step is to identify the target vein and adjacent anatomical structures, using short-axis (transverse) and long-axis (longitudinal) view ultrasound [25]. This requires the operator's visual focus to switch between ultrasound screen and patient, using both short-axis and long-axis views to verify target vessels, while remembering at least 10 images simultaneously. Moreover, operators need to coordinate their hands and eyes to puncture the target vein while simultaneously tracking both image and patient. Accurately visualizing anatomic structures from 2D images and mustering the necessary eye-hand coordination and dexterity imposes significant cognitive load [25, 26]. In traditional ultrasound training, learners are typically required to monitor the ultrasound screen while simultaneously observing the patient's anatomical landmarks. For novice physicians, this dual tasking produces an excessive amount of information to process concurrently, thereby leading to increased cognitive load. To address this issue, we aim to introduce AR to assist in learning by reducing the cognitive load on trainees. AR technology enables a more accurate and intuitive image display, allowing operators to view ultrasound image and patient within the same frame of view in real-time. This study investigated the application of AR technology during skill acquisition by trainee physicians rehearsing US-guided intervention procedures for CVC placement. We compared the performance and cognitive load of trainees using ultrasound imaging displayed through AR head-mounted displays (AR-US) with trainees using standard ultrasound screens (S-US). We hypothesized that AR applications aiming to optimize cognitive load by integrating real and virtual elements, would enhance conceptual learning through their multiple co-located representations. The split-attention effect suggests that retrieving information from separate external sources (e.g. ultrasound screens) increases extraneous cognitive load, while retrieving information from temporally and spatially integrated sources, such as real-world objects (e.g. simulators) augmented with overlaid representations (e.g. ultrasound images on AR-HMDs), would reduce the extraneous cognitive load.

## Methods

### Study design

This multicenter, prospective, randomized crossover study used data acquired in three distinct simulation facilities in northern Taiwan, from September 1st, 2021, through December 31st, 2022. The trial was registered with ClinicalTrials.gov on 20/09/2023, and Supplement 1 provides a comprehensive study protocol. The study followed the CONSORT reporting criteria (Supplement 2, eTable 1). Ethical approval was granted by the local ethics committee, and informed written consent, including consent for the publication of their images, was obtained from all participants.

**Fig. 1:**
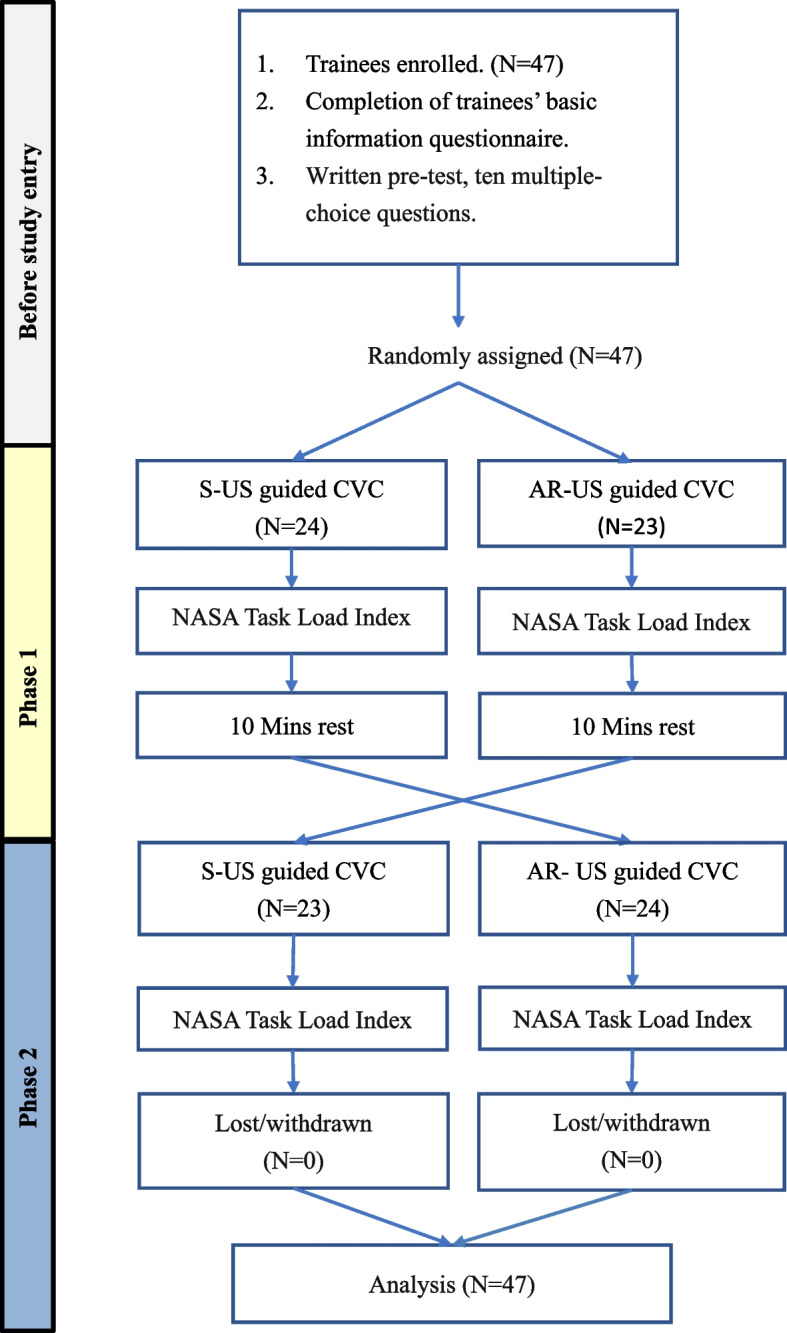
Study flowchart. CVC: Central venous catheter. AR-US: Augmented reality ultrasound using a head mounted display. S-US: Standard ultrasound without visual overlay

Figure 1 shows the 3-stage experiment. In the baseline phase, all trainees completed a written pre-test, ten multiple-choice questions to evaluate their baseline knowledge, and a questionnaire on basic information and past experience with US-guided CVC procedures. In Phase 1, trainees were randomly assigned using a standard back-of-the-hand fair coin-toss method to join either the S-US or AR-US group, with "Heads" assigning them to the AR-US group for the Phase 1 round and “Tails” assigning them to the S-US group for the Phase 1 round. Any poorly performed or questionable coin-toss operations were simply disregarded and repeated. During the trial, the AR-US group wore AR-HMDs, while the S-US group wore head-mounted cameras. Both groups then performed a CVC placement simulation exercise using the appropriate instruments and subsequently completed the NASA Task Load Index (NASA-TLX). The subjects were not blinded during this trial.

In Phase 2, the trainees switched their randomly assigned US modality, repeated the CVC procedures and again completed the NASA-TLX.

Since the participants had no prior experience using AR-US, their unfamiliarity with the AR-HMDs could potentially act as a confounder. Therefore, before participating in the trial, the trainees were given a ten-minute acclimation period with the AR-HMDs in the simulation lab. During this period, they familiarized themselves with the AR-HMD's display of ultrasound images using a different ultrasound simulator (CAE Blue Phantom Select Series Ultrasound Branched 4-Vessel Training Block).

Given the lack of prior research on the application of AR technology in CVC placement, we hypothesized a 15-s difference in ultrasound usage time (TU) between the AR-US group and the S-US group. Additionally, we postulated a 5-point difference in the NASA-TLX scores between the two groups. By applying the formula N = (Z_(α/2) × σ)/E (alpha = 0.05), we determined the required sample sizes for the AR-US and S-US groups to be 48 and 56 participants, respectively.

### Study population

In Taiwan, formal medical education involves six years of undergraduate training (UGY), followed by national licensing exams. Subsequently, licensed physicians must undertake a two-year postgraduate training (PGY) program in general medicine before selecting their specialized residency [27]. We recruited final year UGY students and PGY physicians from three hospitals in Taiwan. All participants had to complete training in US-guided CVC placement two weeks before the study, covering point-of-care ultrasound training (3 h) and US-guided CVC placement training (2 h), using a simulator. None had previous experience with AR in US-guided CVC placement in real patients.

### Interventions

**Fig. 2:**
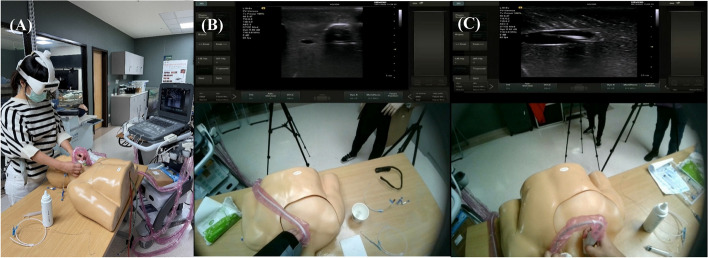
Practical operation. **A** Trainee actively engaged in the process of executing AR-US. **B** and **C** Visual perspectives observed by the trainee using AR-US. AR-US: Augmented reality ultrasound using a head mounted display

We used a SIEMENS ACUSON P500 with linear probe (L10-5v Transducer, 3.5–13.0 MHz) for standard ultrasound (S-US). For AR-US we employed the same probe with customized software to wirelessly transmit ultrasound images to a head-mounted AR display (Foresee-X Smart Surgical Glasses System). During the AR-US procedure, the operator's visual field inside the AR-HMD was split into two parts: the upper part displaying the ultrasound images and the lower part affording a view of the simulator through the glasses (Fig. 2). This enabled the operator to concentrate solely on the intervention field with minimal shifting of visual focus between the ultrasound screen and simulator. The basic setup of the Foresee-X Smart Surgical Glasses system consists of an ultrasound device, a control system, and an AR-HMD. The control system is a signal processing unit that receives signals from medical imaging devices such as C-arms, endoscopes, and ultrasound machines, and displays them on both the AR headset and a monitor. This system supports various signal inputs, including Video Graphics Adapter (VGA), Bayonet Neill-Concelman (BNC), and Digital Visual Interface (DVI), and can connect to any medical imaging device [3, 28]. (Supplement 2, eFigure 1).

### Task simulator

Trainees practiced CVC placement on a task trainer (Blue Phantom®, Gen II Ultrasound Central Line Training Model, CAE Healthcare, Sarasota, FL). Upon completion, research staff examined the blood volume within the simulator vessel, replenished it and removed any excess air to ensure identical testing conditions for the next trainee**.**

### Outcome measurements

#### US-guided CVC placement performance

Quantitative data collected during specific stages of the procedure [29], as measured through video recordings included: T_1_, time required using ultrasound to identify the anatomy of the insertion site and locate the internal jugular vein (IJV); T_2_, time required using real-time ultrasound guidance to puncture the IJV with a CVC syringe (Seldinger needle) and obtain venous flashback; T_U_, the sum of T_1_ and T_2_, i.e., aggregate duration of ultrasound employment; T_NU_, duration of guide wire insertion and CVC placement, i.e. time required without the aid of ultrasound during the procedure. Other data included: number of attempts needed to puncture the vein, frequency of visual focus shift from ultrasound display to simulator, first pass rate, and a score used to assess trainees’ US-guided intervention skills.

#### Scoring system

The scoring system assessed the trainees’ proficiency in CVC placement by addressing six specific components to evaluate technical abilities [30]. These included: vessel identification, needle puncture along the probe axis, needle visualization, needle tip visualization, IJV puncture with venous return and guide insertion. The three-level scoring criteria assessed the level of assistance required from the instructor during execution of these skills:

- Level 1: Instructor had to provide hands-on help: Trainees required direct physical assistance from the instructor to successfully complete the procedure.
- Level 2: Instructor had to provide verbal instruction: Trainees needed guidance and instruction from the instructor, but not direct physical assistance.
- Level 3: The procedure was performed without help: Trainees successfully executed the procedure without assistance.

#### Cognitive load measurement

The level of cognitive load often significantly affects learning outcomes. Numerous studies have utilized the NASA-TLX to measure learning effectiveness when comparing innovative teaching methods, such as AR, virtual reality and extended reality, with traditional instructional materials. This tool has been widely validated across various contexts [31–37]. The NASA-TLX assesses the perceived mental workload of subjects across six dimensions. Three dimensions involve demands placed on the subject (Mental-, Physical- and Temporal Demands), while the other three involve interaction between subject and task (Effort, Frustration and Performance). Subjects assign NASA-TLX numerical ratings to each dimension to evaluate their workload by marking the desired location on a 12 cm line divided into 20 intervals, representing 20 points between bipolar anchoring descriptors which denote low and high cognitive load levels [38].

### Statistical analysis

Continuous variables were presented as mean (SD) or median (Q1-Q3); categorical variables were presented as count (%). Continuous variables were compared using Student’s t-test or Wilcoxon test for independent groups, and paired t-test for dependent groups. Categorical variables were compared using Chi-square test. Kendall rank correlation coefficient was used to observe correlation between variables. We compared T_1_, T_2_, T_U_ and T_NU_ between the two arms to evaluate the trainees’ ultrasound-guided intervention skills. We compared the NASA-TLX scores to evaluate cognitive workload and usability in ultrasound-guided CVC placement. To explore possible factors affecting the outcome measures, we conducted subgroup analysis by seniority and sex. Due to concerns that the crossover design might result in a learning effect during the second CVC placement process, regardless of the order in which AR-US and S-US were assigned, we evaluated potential carry-over effects by comparing the results from Phase 1 and Phase 2 for each participant to assess their performance in CVC placement. We considered a *p*-value of < 0.05 as statistically significant. The statistical analysis was performed using SAS version 9.4 (SAS Institute, Cary, NC, USA)**.**

## Results

### Characteristics of participants

This trial initially aimed to enroll 56 participants. However, due to the COVID-19 pandemic, many potential participants were unable to attend the trial as scheduled, resulting in a final sample size of 47 participants, with 32 males (68%) and 15 females (32%). These included 22 final-year UGY students and 25 postgraduate year 1–4 residents. Most trainees (41/47, 87.2%) had prior experience with CVC placement using ultrasound on simulators. However, only half (20/47, 42.6%) had experience with CVC placement on real patients using landmark guidance, and only a quarter (14/47, 29.8%) had experience with CVC placement on real patients using ultrasound guidance (Table 1).

**Table 1:** Trainee characteristics: Descriptive analysis of trainee demographics, experience and prior knowledge of ultrasound-guided CVC placement

Total trainees, N=47	
Male / Female, N (%)	32 / 15 (68.1%, 31.9%)
Age, mean+SD	28.15+3.39
Female	27.13+3.66
Male	28.63+3.18
UGY, N (%)	22 (46.8%)
PGY1-2, in general medicine, N (%)	15 (31.9%)
PGY3-4, in specialty, N (%)	10 (21.3%)
Emergency medicine	3 (6.4%)
Anesthesiology	1 (2.1%)
Surgical specialties	4 (8.5%)
Neurology	2 (4.3%)
CVC placement experience, N (%)	
Practice on simulator, US-guided	41 (87.2%)
Practice on real patient, landmark guided	20 (42.6%)
Practice on real patient, US-guided	14 (29.8%)

*CVC* Central venous catheter, *UGY* Undergraduate medical students, *PGY *Postgraduate physicians, *US *Ultrasound

### Performance of ultrasound guided intervention skills

**Fig. 3:**
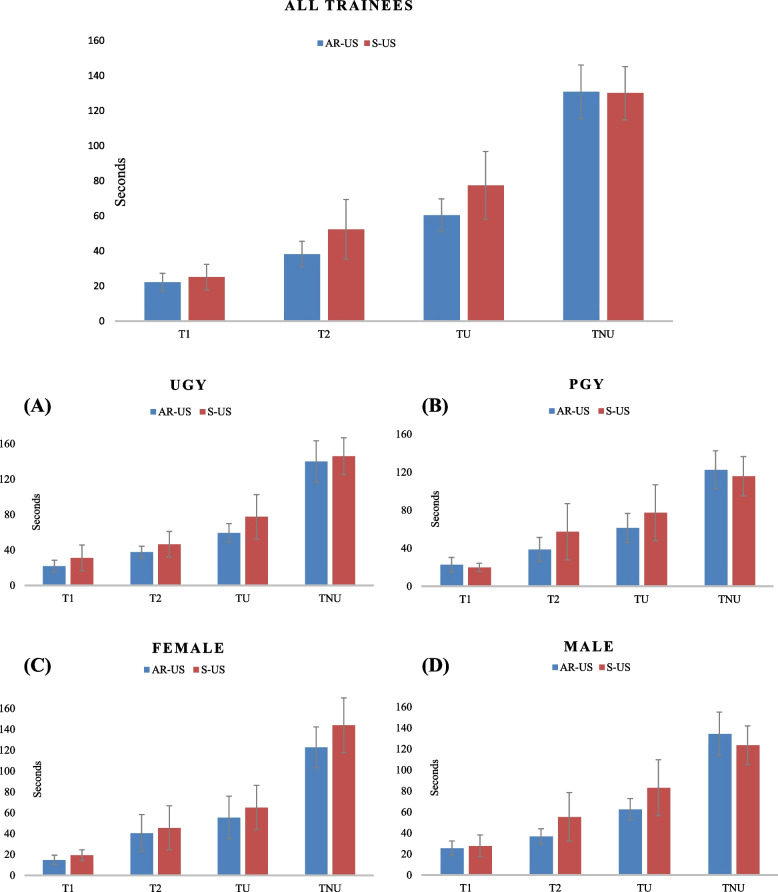
Comparing the trainees' ultrasound-guided intervention skills using AR-US and S-US. **A** and **B** The performance of trainees with different seniority, with AR-US and S-US. **C** and **D** The performance of trainees of different gender, with AR-US and S-US. T_1_: Time required using ultrasound to identify the anatomy of the insertion site and locate IJV. T_2_: Time required under real-time ultrasound guidance to puncture IJV with a CVC syringe (Seldinger needle) and obtain venous flashback. T_U_ = T_1_ + T_2_: Procedure time with US. T_NU_: Duration of guide wire insertion and CVC placement during the procedure. Specifically, time required to complete this process without the aid of ultrasound. CVC: Central venous catheter. UGY: Undergraduate students. PGY: Postgraduate physicians. AR-US: Augmented reality ultrasound using a head mounted display. S-US: Standard ultrasound without visual overlay

Comparing AR-US vs. S-US among all trainees, the times spent on each step of the procedure were as follows: T_1_ (seconds): 22.21 (17.77) vs. 25.11 (25.51), mean difference: -2.90 (95% CI: -11.91 to 6.13), *p* = 0.53; T_2_ (seconds): 38.19 (25.54) vs. 52.32 (59.60), mean difference: -14.13 (95% CI: -33.03 to 4.78), *p* = 0.14; T_U_(seconds): 60.40 (32.82) vs. 77.43 (67.67), mean difference: -17.03 (95% CI: -38.92 to 4.88), *p* = 0.13 (Table 2). We found the times T_1_, T_2_ and T_U_ to be shorter for AR-US than for S-US, although without reaching statistical significance (Fig. 3), which may be attributed to the limited number of participants and the varying levels of experience with both ultrasound and AR. However, there was a significant difference between AR-US and S-US in the frequency of visual focus shift from US display to simulator (1 vs. 5, *p* < 0.001) (Table 3).

**Table 2:** Comparison of trainee performance in AR-US and S-US groups for CVC placement

	**AR-US, seconds**	**S-US, seconds**	**Mean difference (95% CI)**	***p*-value**
**T**_**1**_ (N)	**Mean (SD)**	**Mean (SD)**		
All (47)	22.21 (17.77)	25.11 (25.51)	-2.90 (-11.91, 6.13)	0.53
UGY (22)	21.77 (15.94)	31.05 (34.92)	-9.28 (-26.00, 7.45)	0.27
PGY (25)	22.60 (19.56)	19.88 (10.94)	2.72 (-6.36, 11.80)	0.55
Male (32)	25.66 (19.89)	27.75 (29.95)	-2.09 (-14.84, 10.65)	0.74
Female (15)	14.87 (8.75)	19.47 (10.10)	-4.60 (-11.67, 2.47)	0.19
**T**_**2**_ (N)	**Mean (SD)**	**Mean (SD)**		
All (47)	38.19 (25.54)	52.32 (59.60)	-14.13 (-33.03, 4.78)	0.14
UGY (22)	37.59 (15.86)	46.50 (34.82)	-8.91 (-25.59, 7.77)	0.28
PGY (25)	38.72 (32.08)	57.44 (75.42)	-18.72 (-52.09, 14.65)	0.26
Male (32)	37.06 (20.30)	55.44 (66.74)	-18.38 (-43.37, 6.62)	0.14
Female (15)	40.60 (34.94)	45.67 (41.70)	-5.07 (-33.88, 23.71)	0.72
**T**_**U**_ (N)	**Mean (SD)**	**Mean (SD)**		
All (47)	60.40 (32.82)	77.43 (67.67)	-17.03 (-38.92, 4.88)	0.13
UGY (22)	59.36 (24.86)	77.55 (60.01)	-18.19 (-46.55, 10.19)	0.20
PGY (25)	61.32 (39.01)	77.32 (75.01)	-16.00 (-50.29, 18.29)	0.35
Male (32)	62.72 (29.14)	83.19 (76.80)	-20.47 (-49.82, 8.88)	0.17
Female (15)	55.47 (40.26)	65.13 (41.83)	-9.66 (-40.37, 21.04)	0.52
**T**_**NU**_ (N)	**Mean (SD)**	**Mean (SD)**		
All (47)	130.70 (53.64)	130.00 (53.01)	0**.**70 (-21.12, 22.57)	0.95
UGY (22)	140.05 (56.11)	146.05 (49.96)	-6.00 (-38.32, 26.32)	0.71
PGY (25)	122.50 (51.09)	115.84 (52.50)	6.66 (-22.82, 36.10)	0.65
Male (32)	134.44 (59.55)	123.50 (53.09)	10.94 (-17.26, 39.13)	0.44
Female (15)	122.73 (38.76)	143.80 (51.86)	-21.07 (-55.31, 13.17)	0.22

T_1_Time required to use ultrasound to identify the anatomy of the insertion site and locate IJV

T_2_Time required under real-time ultrasound guidance to puncture IJV with a CVC syringe (Seldinger needle) and obtain venous flashback

T_U_T1 + T2: Procedure time using US

T_NU_Duration of guide wire insertion and CVC placement during the procedure. Specifically, the time required to complete this process without the aid of ultrasound

*CVC* Central venous catheter, *UGY *Undergraduate students, *PGY *Postgraduate physicians, *AR-USA* Ugmented reality ultrasound using a head mounted display, *S-US* Standard ultrasound without visual overlay

**Table 3:** Comparison of differences in AR-US and S-US groups for CVC placement

	**AR-US**	**S-US**	***p*-value**
**Puncture attempt number** (N)	**Median (Q1-Q3)**	**Median (Q1-Q3)**	
All (47)	1(1–2)	1(1–2)	0.43
UGY (22)	1(1–3)	1(1–2)	0.28
PGY (25)	1(1–2)	1(1–2)	0.94
Male (32)	2(1–3)	1(1–2)	0.07
Female (15)	1(1–1)	1(1–3)	0.24
**Frequency of visual focus shift from US display to simulator** (N)	**Median (Q1-Q3)**	**Median (Q1-Q3)**	
All (47)	1 (0–2)	5 (4–7)	< 0.01^*^
UGY (22)	1 (0–1)	5 (4–7)	< 0.01^*^
PGY (25)	1 (0–2)	5 (3–7)	< 0.01^*^
Male (32)	0 (0–1)	5 (4–11.5)	< 0.01^*^
Female (15)	1 (0–3)	5 (3–6)	< 0.01^*^
**First pass rate** (%)	**N (%)**	**N (%)**	
All (47)	35 (74.47)	31 (65.96)	0.50^†^
UGY (22)	15 (68.18)	17 (77.27)	0.74^†^
PGY (25)	20 (80)	14 (56)	0.13^†^
Male (32)	25 (78.13)	22 (68.75)	0.57^†^
Female (15)	10 (66.67)	9 (60)	1^†^

*CVC* Central venous catheter, *UGY* Undergraduate students, *PGY* Postgraduate physicians, *AR-US* Augmented reality ultrasound using a head mounted display, *S-US* Standard ultrasound without visual overlay

*p-value < 0.05

^†^Chi-squared test

**Fig. 4:**
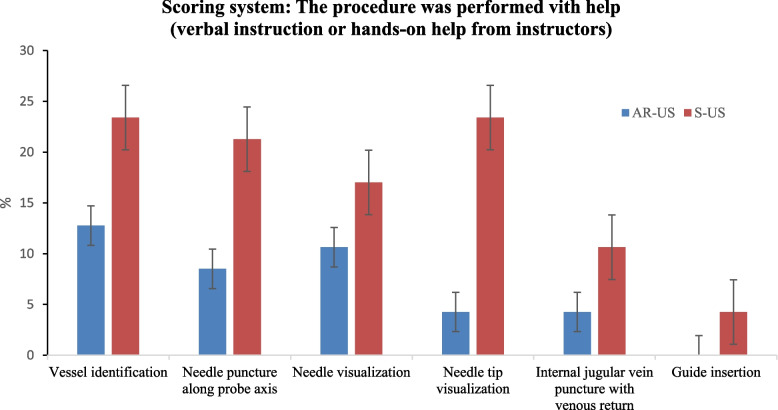
Scoring system to evaluate trainees’ ultrasound guided intervention skills. AR-US: Augmented reality ultrasound using a head mounted display. S-US: Standard ultrasound without visual overlay

The scoring system assessed the degree of instructor assistance required during CVC placement. For each step, approximately 10% of the AR-US trainees needed assistance, but not hands-on help from the instructor. By contrast, a higher proportion of the S-US group, around 15%, needed assistance, including hands-on instructor guidance in every step. This difference was particularly evident in the needle tip visualization step (AR-US vs. S-US: 4.26% vs. 10.64%), a task that demands significant hand–eye coordination. Trainees using standard ultrasound had to maneuver the CVC needle and syringe within a very confined surgical area while simultaneously shifting their gaze between the ultrasound screen and the surgical field. This frequent visual transition often resulted in a loss of orientation. Notably, the AR-US group exhibited a higher level of independence in successfully completing ultrasound operations (Fig. 4).

### Cognitive workload and usability

**Fig. 5:**
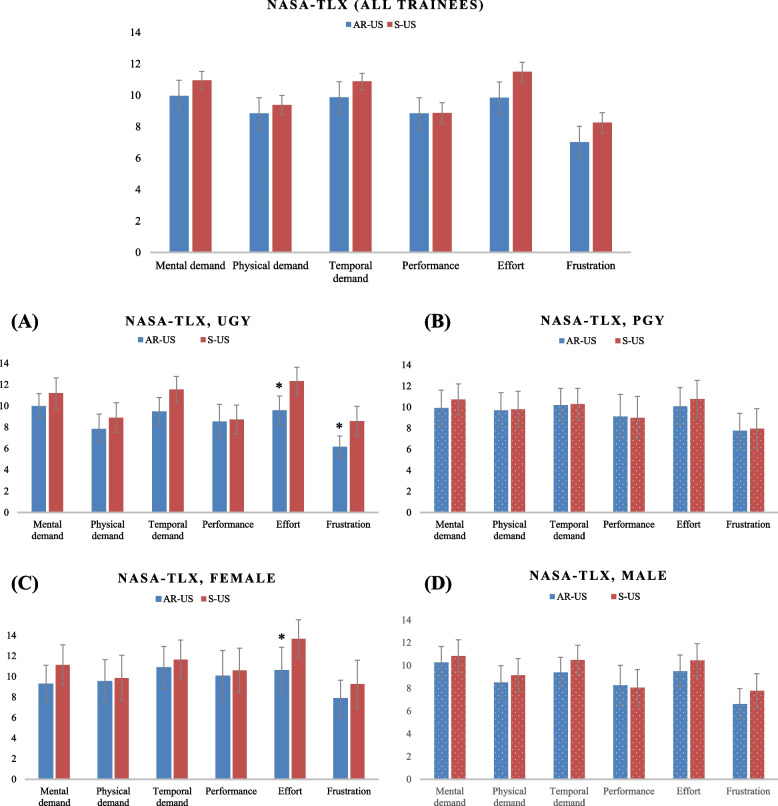
Comparison of NASA-TLX scores between AR-US and S-US groups: A measure of cognitive workload and usability in ultrasound-guided CVC placement. **A** and **B** The NASA-TLX scores of trainees with different seniority, with AR-US and S-US. **C** and **D** The NASA-TLX scores of trainees of different gender, with AR-US and S-US. NASA-TLX: NASA Task Load Index. CVC: Central venous catheter. UGY: Undergraduate students. PGY: Postgraduate physicians. AR-US: Augmented reality ultrasound using a head mounted display. S-US: Standard ultrasound without visual overlay. *p-value < 0.05

Among all trainees, scores in the six NASA-TLX dimensions for the AR-US vs. S-US groups were as follows: Mental demand: 9.96 (3.84) vs. 10.94 (3.92), mean difference: -0.98 (95% CI: -2.57 to 0.61), *p* = 0.22; Physical demand: 8.85 (4.13) vs. 9.38 (4.17), mean difference: -0.53 (95% CI: -2.23 to 1.17), *p* = 0.54; Temporal demand: 9.87 (3.86) vs. 10.87 (3.66), mean difference: -1.00 (95% CI: -2.54 to 0.54), *p* = 0.20; Performance: 8.85 (4.95) vs. 8.87 (4.53), mean difference: -0.02 (95% CI: -1.97 to 1.92), *p* = 0.98; Effort: 9.85 (4.16) vs. 11.49 (4.23), mean difference: -1.64 (95% CI: -3.36 to 0.08), *p* = 0.06; Frustration: 7.02 (3.70) vs. 8.26 (4.38), mean difference: -1.24 (95% CI: -2.89 to 0.43), *p* = 0.14. We found the AR-US group had lower scores than the S-US group for most dimensions, except performance, indicating a comparatively lower perceived mental workload among trainees using AR technology (Fig. 5).

### Subgroup analysis

In the subgroup analysis in both groups we observed no differences, based on seniority and gender, in the time taken for each step of CVC placement. However, consistent with the main analysis including all participants, we noticed that the AR-US group tended to spend less time on T_1_, T_2_ and T_U_, compared to the S-US group, in the different gender and seniority subgroups (Figs. 3A, 3B, 3C, 3D). Also, we found significant differences in the NASA-TLX scores between the AR-US and S-US groups. Specifically, junior trainees using AR-US, compared to S-US, had lower scores for effort [9.59 (3.85) vs. 12.32 (3.76), mean difference: -2.73 (95% CI: -5.04 to -0.41), *p* = 0.02] and frustration [6.18 (2.89) vs. 8.59 (3.94), mean difference: -2.41 (95% CI: -4.51 to -0.31), *p* = 0.03] (Fig. 5A). Among female trainees, the NASA-TLX scores for effort were lower for AR-US, compared to S-US [10.6 (4.44) vs. 13.67 (3.64), mean difference: -3.07 (95% CI: -6.10 to -0.03), *p* = 0.05] (Fig. 5C).

**Fig. 6:**
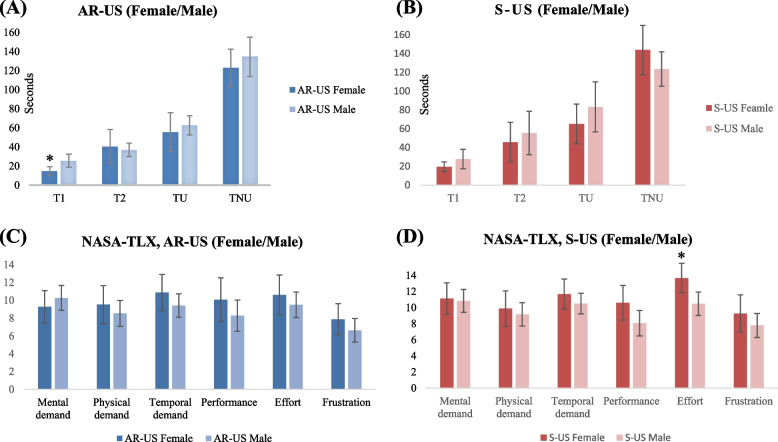
Comparing the performance and NASA-TLX of different genders using AR-US and S-US. **A** The performance of participants of different gender in the AR-US group. **B** The performance of participants of different gender in S-US group. **C** The NASA Task Load Index (NASA-TLX) scores of participants of different gender using AR-US. **D** The NASA Task Load Index (NASA-TLX) scores of participants of different gender using S-US. T_1_: Time required using ultrasound to identify the anatomy of the insertion site and locate IJV. T_2_: Time required under real-time ultrasound guidance to puncture IJV with a CVC syringe (Seldinger needle) and obtain venous flashback. T_U_ = T_1_ + T_2_: Procedure time with US. T_NU_: Duration of guide wire insertion and CVC placement during the procedure. Specifically, time required to complete this process without the aid of ultrasound. AR-US: Augmented reality ultrasound using a head mounted display. S-US: Standard ultrasound without visual overlay. *p-value < 0.05

Comparing performance and NASA-TLX of females and males using the same ultrasound modality, we observed that female trainees using AR-US, compared to male trainees using AR-US, were able to identify the anatomy of the insertion site and locate the IJV in less time (T_1_(s): 14.87 (8.75) vs. 25.66 (19.89), mean difference: -10.79 (95% CI: -19.21 to -2.37), p = 0.01) (Fig. 6A). Additionally, we found that female trainees using S-US recorded a significantly higher NASA-TLX effort score than male trainees did (13.67 (3.64) vs. 10.47 (4.14), mean difference: 3.20 (95% CI: 0.77 to 5.63), p = 0.01) (Fig. 6D).

### Validation of learning and carry-over effects

We found no significant difference between the Phase 1 and Phase 2 results for most time intervals, suggesting no carry-over effects, except in T_NU_, where no ultrasound assistance was actually required (Supplement 2, eTable 2).

## Discussion

This study was the first to investigate the acquisition of skills by trainees applying AR technology in ultrasound-guided interventions for CVC placement. We examined the effectiveness of AR by evaluating trainees' performance and cognitive load. We found that AR technology reduced the time required for critical steps, minimized neck strain for users, and assisted trainees in performing ultrasound-guided CVC placement. AR technology also reduced trainees’ dependence on instructor assistance. Furthermore, participants perceived lower mental and physical burdens using AR-US, compared to S-US, to achieve the same performance level. These effects of AR were especially observed in junior- and female trainees, suggesting that AR technology may be a useful tool to enhance medical training in the field of CVC placement for UGY students and PGY physicians.

Our results showed that both T_1_ and T_2_ times were shorter with AR-US, compared to S-US. T_1_ reduction was achieved by projecting the ultrasound image onto the head-mounted AR-display, enabling the user to merge their view of the operative field with the ultrasound image, thereby reducing extraneous cognitive load. T_2_ involved both ultrasound guidance and eye-hand coordination, increasing both extraneous and intrinsic cognitive load due to the difficulty of the task. The observed T_2_ performance improvement with AR may be related to the interplay of sensory memory, working memory and long-term memory during the learning process. Sensory memory is of large capacity but short retention time. Working memory has limited capacity and also brief retention. To work within the limitations of working memory, learners must organize information into meaningful chunks and connect it to relevant prior knowledge in long-term memory. These capacity constraints of working memory hinder simultaneous retention of large amounts of information, making it a bottleneck in the learning process [39–41]. Cognitive Load Theory distinguishes between intrinsic (required), extraneous (required) and germane cognitive load (resulting from the learner's deliberate use of cognitive strategies to improve learning, like creating mental structures) [42]. When cognitive load exceeds working memory capacity, learning and performance may suffer [41–43]. AR technology can reduce external load by integrating the US image and simulator into the same field of view, thereby reducing the amount of real-time information retained in working memory, and enhancing trainees’ performance in CVC placement.

Several studies have shown that AR-US can be beneficial during biopsy and nerve block procedures. Kasuya Y. et al. included eight experienced anesthesiologists who performed ultrasound-guided peripheral nerve blocks on a training simulator. They found that using an AR-HMD reduced procedure time and improved needle visibility on ultrasound [44]. Shimizu T. et al. found that AR-US significantly reduced rates of unintentional puncture of non-target lesions during needle puncture procedures. AR-US significantly reduced insertion and advancement of the needle beyond the target lesion, compared to S-US [45]. Our study found AR may help trainees to manage multiple tasks simultaneously, including searching for target vessels, memorizing target vein location on the ultrasound screen, and recognizing surrounding anatomical structures such as sternocleidomastoid muscle, trachea, thyroid gland and common carotid artery, and facilitate shifting the gaze to compare with relevant locations on the simulator. The ultrasound screen should ideally be positioned directly in the practitioner's line of vision to maintain a straight, ergonomic alignment of the visual and motor axes, eliminating the need to raise and lower the operator’s head to alternately focus on the vessels displayed on screen and the corresponding simulator locations. In this way, AR can facilitate accurate and detailed representation of spatial information while reducing extraneous cognitive load and improving learning efficiency [12, 46].

Two theories derived from Cognitive Load Theory—the Cognitive Theory of Multimedia Learning (CTML) [47] and the Cognitive-Affective Theory of Learning with Media (CATLM) [48]—also support the notion that the integration of AR can enhance learning outcomes. CTML posits that learning is more effective when information is presented through both visual and verbal channels, aiding the integration of new knowledge into existing cognitive frameworks. Traditional ultrasound training requires learners to divide their attention between the ultrasound screen and the patient's anatomical landmarks, leading to a high cognitive load that can impede learning. AR technology can address this issue by overlaying relevant information directly onto the learner's field of view, thereby minimizing the need for cognitive switching and facilitating a more seamless integration of visual and verbal information. This approach can reduce external load by combining the ultrasound image and simulator into the same visual field, decreasing the real-time information burden on working memory and enhancing trainees’ performance in central venous catheter (CVC) placement. This alignment with CTML principles indicates that AR has the potential to significantly improve the learning process by optimizing cognitive load management. CATLM extends this perspective by highlighting the importance of emotional and motivational factors in media-based learning. This theory suggests that learning is not solely a cognitive process but also an affective one, where emotional engagement and motivation play crucial roles. AR technology can make the learning experience more interactive and engaging, thereby boosting motivation and reducing frustration. By providing real-time feedback and visual guidance, AR can enhance learners' confidence and competence, leading to better retention and transfer of skills to clinical practice.

We found that female trainees largely had better outcomes using AR-US than male trainees during T_1_, which mainly involved recognition of the insertion site anatomy and target vessel location. Hunter-gatherer theory [49] offers a unique perspective for this observation, postulating that females perform better than males in object location memory, while males perform better in three-dimensional mental rotations. AR potentially taps into this superior visual-spatial working memory of female trainees. In line with these AR advantages for female trainees, the NASA-TLX analysis also showed that females using S-US reported significantly higher scores than males for "effort to accomplish the level of performance", indicating that females experience greater psychological and physiological stress when using standard US to complete tasks. Amir Dirin et al. conducted a study to investigate the impact of various instructional materials on learners' emotional responses during the learning process. The findings indicate that videos, virtual reality (VR), and AR elicited more positive emotional responses in females, compared to males. Emotional response to new technology is crucial for its adoption across various applications. Furthermore, the results suggest that females prefer audio-visual and interactive technologies. This positive emotional response, coupled with AR's ability to reduce extraneous cognitive load, may also explain why female learners tend to perform better [50].

We also found that junior trainees tended to have superior performance using AR-US, and their experiencing less effort and frustration could be attributed to their lack of relevant experience and attendant expectations, compared with more experienced physicians. As novice physicians, they are more adaptable and open to new technologies, and less vulnerable to pre-existing biases or habits that may hinder their learning. More experienced physicians with specific skills may find it more challenging to adapt to new technologies that challenge their established knowledge and practices [51]. These findings suggest that introducing AR-US to medical education at an early stage, such as during clerkship, could be beneficial as junior learners are more receptive to new technologies and more accustomed to new learning strategies.

AR is widely considered advantageous for the acquisition of ultrasound-guided procedural skills, as the integration of real-time, high-resolution ultrasound images into the visual field significantly enhances ergonomic efficiency by minimizing distractions. This allows the performing physician to maintain focus on both the ultrasound image and the operational field concurrently, thereby eliminating the need to alternate attention between these two critical areas. However, some uncertainty remains about whether AR is a revolutionary tool for medical assistance or merely a novelty. Empirical evidence presents a nuanced perspective. Some studies indicate that AR does not significantly improve the proficiency of practitioners in performing ultrasound-guided biopsies, despite the subjective preference for AR as a more engaging instructional modality. Additionally, AR has been associated with a steeper learning curve in these procedures, which may be attributed to the learners' unfamiliarity with AR devices [50, 52, 53]. In a recent randomized clinical trial concerning ultrasound-guided radial artery catheterization in pediatric patients, AR-HMD demonstrated superior efficacy in training junior anesthesia residents but did not yield comparable benefits for senior pediatric anesthesiology fellows [11]. These findings underscore the necessity for more extensive, well-powered studies to investigate the efficacy of AR-HMD in enhancing ultrasound-guided procedural training across various levels of clinical experience.

This study has several limitations. First, the target of the US-guided intervention in this study was a CVC simulator, not a patient. Simulators have no anatomical variations like real humans. Further research is needed to apply these findings to real patients. Moreover, the practitioners participating in this study had no experience using AR-HMDs. Therefore, their performance may have been affected by unfamiliarity with the AR-HMD hardware, leading to errors or even increased cognitive load. Furthermore, the ease with which trainees can adapt to AR technology largely depends on their prior experience using AR-HMDs and other complex devices, such as smartphones, tablets, smart glasses and virtual reality headsets. To address these three limitations of the study, we applied a randomized crossover design to minimize potential effects of biases. To mitigate the unavoidable carry-over effects in a crossover design, we compared the Phase 1 and Phase 2 results, whereby we found no significant differences in times, suggesting that the carry-over effects were negligible (Supplement 2, eTable 2).

Technological innovations like artificial intelligence, VR, AR and mixed reality (MR) are becoming integral to our daily and professional lives. In critical medicine, AR has been utilized for vascular access, biopsy procedures, and educational purposes. Future research should investigate the use of AR in combination with imaging techniques, such as ultrasound, in clinical settings. Moreover, studies should examine the effects of AR-assisted methods on patient safety and evaluate the long-term learning outcomes and retention for medical trainees.

## Conclusion

The application of AR technology during skill acquisition helps reduce cognitive load and improves trainees' performance in ultrasound-guided interventions for CVC placement, especially by reducing the time required for critical steps, minimizing neck strain and assisting operators. Our results also indicated that AR technology could help reduce trainees’ mental workload and dependence on instructors. Our findings suggested that AR technology may be a useful tool to enhance medical training in the field of CVC placement for UGY medical students and PGY physicians.

Given the widespread adoption of POCUS in clinical practice and the increasing portability and affordability of ultrasound machines, various ultrasound societies now recommend incorporating POCUS training into undergraduate curricula. Although POCUS training is increasingly promoted at the undergraduate level, UGYs and novice physicians often lack familiarity with medical knowledge and clinical applications. This study employed AR technology to reduce cognitive load, helping young trainees overcome learning anxiety and advancing future POCUS education.

Although the study's findings are encouraging, further research involving real patients instead of simulators is needed to validate the effectiveness of AR in clinical settings. Additionally, long-term studies examining skill retention and the transfer of skills to clinical practice could offer deeper insights into the potential benefits of AR technology in medical education.

## Supplementary Information

Supplementary Material 1.

Supplementary Material 2.

## Data Availability

All relevant original data is available from the corresponding author on reasonable request.

## Abbreviations

AR: Augmented reality
AR-US: Augmented reality ultrasound using a head mounted display.
CVC: Central venous catheter
HMD: Head-mounted display
IJV: Internal jugular vein
NASA-TLX: NASA Task Load Index
PGY: Postgraduate training
POCUS: Point-of-care ultrasound
S-US: Standard ultrasound
UGY: Undergraduate training
VR: Virtual reality

## Data availability

Data is provided within the manuscript or supplementary information files.
