# Supplementary material for: Augmented reality visualization for ultrasound-guided interventions: a pilot randomized crossover trial to assess trainee performance and cognitive load
Source: BMC Med Educ. 2024 Sep 27;24:1058. doi: 10.1186/s12909-024-05998-8 (PMC11429828; doi:10.1186/s12909-024-05998-8)
Supplement: Supplementary file 1 — Supplementary Material 1. [file 12909_2024_5998_MOESM1_ESM.docx]

**Study Protocol and Statistical Analysis Plan**

**Title:** Augmented Reality Simulation for Invasive Procedure Training: An

investigation of medical students’ and trainees’ performance and

cognitive load during skill acquisition using AR head-mounted display.

Effect of quality assured simulation training on cardiac device implanter skills

**Principal Investigator:**

Shu-Chen Liao: Department of Emergency Medicine, Keelung Chang Gung Memorial Hospital, Keelung, Taiwan. (ermdsusan@gmail.com)

**Co-investigators:**

Yu-Che Chang: Department of Emergency Medicine, Linkou Chang Gung Memorial Hospital, Taoyuan, Taiwan.

Chung-Hsien Chaou: Department of Emergency Medicine, Linkou Chang Gung Memorial Hospital, Taoyuan, Taiwan.

Shih-Chieh Shao: Department of Pharmacy, Keelung Chang Gung Memorial Hospital, Keelung, Taiwan

**Sponsor:**

None

**Funding/Support:**

The work was funded by Ministry of Science and Technology, Taiwan (NMRPD1L1131).

Taiwan Main Orthopedic Biotechnology Company will support the acquisition of Foresee-X Smart Surgical Glasses for simulation training. Taiwan Main Orthopedic Biotechnology Company did not and will not influence design and conduct of the study; collection, management, analysis and interpretation of the data; preparation, review, or approval of any manuscript and decision to submit for publication.

**Version Number**: v.1.0

**Version Date**: 21^st^ December 2020

**Synopsis**

Augmented reality (AR) was initially developed for military purposes and later expanded to everyday life. Currently, AR has found its way into both medical and educational applications.

Central venous catheterization is a vital procedure for managing critically ill patients. Research has confirmed that using real-time ultrasound guidance during the puncture and catheter insertion process substantially enhances its efficacy and safety profile. However, in the standard approach, the use of ultrasound imaging requires that physicians frequently switch focus between the ultrasound monitor and the procedural site. This constant shifting of visual and mental focus intensifies the cognitive burden on the medical professional, leading to "task interruptions and task-switching." Novice physicians attempting to master the CVC procedure may find that this act of task-switching and the ensuing interruptions poses additional, unnecessary challenges, a serious issue that demands greater attention in medical education.

Taking the placement of a central venous catheter as an example, we can now leverage technology to integrate the ultrasound view with the surgical view through the use of AR smart glasses. The ultrasound images are transmitted to the glasses, which also allow a view of the procedure site at the same time, thus reducing the frequent task-switching and its associated cognitive burden. This innovative approach is anticipated to minimize interruptions and transitions during invasive surgeries. We hypothesize that by reducing the cognitive load on the operator, the efficacy of invasive procedures can be enhanced, leading to increased success rates and improved patient safety.

We will conduct a prospective randomized crossover trial to investigate the performance and cognitive load of physicians performing ultrasound-guided central venous catheter (CVC) placement using ultrasound imaging displayed on an AR-enabled head-mounted display (AR-US). The participants will be divided into two groups of 50 each and will take part in a crossover trial involving ultrasound imaging using a traditional monitor and ultrasound imaging using AR glasses. The primary and secondary outcomes will be (1) the number of successful/unsuccessful punctures, (2) the total time taken, and (3) self-assessment of cognitive load. The results of this research as anticipated could be broadly applied to any invasive procedure requiring ultrasound guidance.

Table of Contents

[1. Introduction 4](#_Toc144683271)

[1.1 Background 4](#_Toc144683272)

[1.1.1 Ultrasound-Guided Central Venous Catheter Placement 6](#_Toc144683273)

[1.1.2 Cognitive Load Theory (CLT) 9](#_Toc144683274)

[1.2 Previous Work Justifying this Study 11](#_Toc144683275)

[1.3 Objectives and Hypothesis 12](#_Toc144683276)

[2. Method 14](#_Toc144683277)

[2.1 Existing Central Venous Catheter Training Courses at Chang Gung Hospital 14](#_Toc144683278)

[2.2 Recruitment Criteria 14](#_Toc144683279)

[2.3 Recruitment Methods 14](#_Toc144683280)

[2.4 Sample Size 14](#_Toc144683281)

[2.5 Trial Procedures, Randomization 15](#_Toc144683282)

[2.6 Trial Flowchart 16](#_Toc144683283)

[2.7 Preferred Trial Timetable 17](#_Toc144683284)

[2.8 Task Simulator 17](#_Toc144683285)

[2.9 Smart Glasses 17](#_Toc144683286)

[3. Data Collection 18](#_Toc144683287)

[3.1 Plans for Assessment and Collection of Outcomes 18](#_Toc144683288)

[3.1.1 Characteristics of Participants 18](#_Toc144683289)

[3.1.2 Pre-test 18](#_Toc144683290)

[3.1.3 Primary Outcomes: Performance of CVC Placement 18](#_Toc144683291)

[3.1.4 Secondary outcomes: Cognitive load Evaluation 18](#_Toc144683292)

[3.2 Data Management 18](#_Toc144683293)

[3.3 Confidentiality and Fairness 18](#_Toc144683294)

[3.4 Dissemination 19](#_Toc144683295)

[4. Data Analysis 20](#_Toc144683296)

[5. References 21](#_Toc144683297)

# Introduction

# Background

With rapid advancements in technology, the application fields of virtual reality (VR) and augmented reality (AR) are becoming increasingly broad. Beyond industries like gaming, tourism and entertainment, they have been further extended to education, design, engineering, medicine and nursing in recent years. 'Virtual reality' technology isolates the user from their surrounding environment using VR devices, creating a wholly artificial environment [4]. The presented visuals can be achieved in two ways: one is a simulation of realistic scenes generated entirely by a computer, all of which are virtual; the other is a 360-degree panorama shot, which immerses the user through a head-mounted display. The content captured can be physical. 'Augmented reality', by contrast, refers to overlaying computer-generated virtual objects onto a view of the real world, using tools like smartphones or AR glasses [5]. The concepts of virtual and augmented reality have existed for decades and, with maturing hardware and software, their applications in the medical field are growing daily. According to a report from U.S. medical research, the healthcare market for VR and AR is projected to reach 2.54 billion USD by 2020, with applications spanning diagnosis, surgery, rehabilitation, medical consultation, and educational training.

AR technology has been used increasingly in medical education; recent studies suggest that AR might emerge as a new modality in medical teaching [6]. So far, AR's application in medical education has included interactive scenarios for teaching anatomy, imaging training, and clinical skills [7-9]. However, most such teaching has been assisted by smartphones or tablets. Our research objective focuses on training for invasive clinical procedures common in critical care: specifically, the placement of central venous catheters (CVC) as a case study, exploring whether AR smart glasses can benefit the learning outcomes of the trainees. The planned study will assess the trainees’ cognitive load, the impact of task interruptions, and their ultimate CVC placement performance.

CVC is a type of intravascular catheter placed in large veins, with common placement sites including the internal jugular vein, subclavian vein and femoral vein. CVCs are often used in critically ill patients to provide a pathway for intravenous fluid and specific drug administration. In conditions with significant blood loss, such as gastrointestinal bleeding or trauma, or in emergencies where rapid and large-volume IV fluid is needed, CVCs provide a stable infusion route. Hence, CVC placement may also be recommended before surgeries where there might be significant blood loss. Traditionally, the placement of a CVC is achieved by visualizing or palpating anatomical landmarks [1]. Most practitioners can accomplish the task using these anatomical landmarks, but aberrant anatomy or a prior history of catheter placement can complicate the process, resulting in decreased success rates [2]. Consequently, several guidelines now recommend ultrasound-guided CVC placement [10] (Figure 1).

Figure1. Ultrasound-guided CVC insertion. A), A clinical provider using real-time ultrasound guidance with a high-frequency linear transducer (13-6 MHz) during the insertion of a right internal jugular CVC with a patient in Trendelenburg. B), Transverse view of the right internal jugular vein and the right carotid artery. The clinician can confirm venous puncture using ultrasound, avoiding arterial puncture and iatrogenic pneumothorax [3].

The U.S. National Institute for Clinical Excellence (NICE) recommended in 2002 that ultrasound-guided CVC placement should be the preferred method for elective CVC placement in adults and children [11]. Numerous studies have reported that, compared to the traditional landmark-based approach, ultrasound-guided CVC placement yields similar improvements in success rates and incidence of complications [12, 13]. In a comprehensive meta-analysis conducted in 2013, which included 26 randomized controlled trials (RCTs) involving a total of 4,185 patients (2,081 under ultrasound guidance and 2,104 under landmark guidance), it was found that failures of catheter placement under ultrasound guidance were reduced by 82% (Relative Risk (RR) = 0.18; 95% Confidence Interval (CI): 0.10 – 0.32; p <0.001). Furthermore, significant reductions were observed in complications, including arterial punctures, hematomas, pneumothorax, and hemothorax (p <0.05) [14]. In 2015, another researcher performed an integrated analysis of 17 randomized studies, which included 3,686 adult patients (1,822 under ultrasound guidance and 1,684 under landmark guidance). The findings revealed an increased success rate of 14% for novice physicians (RR = 1.14; 95% CI: 1.08 – 1.2; p <0.001) and a 7% increase for experienced doctors (RR = 1.08; 95% CI: 1.02-1.14; p = 0.011) [15]. The 2015 ACLS and 2017 ATLS guidelines also emphasized that CVC placements are best conducted under ultrasound guidance, primarily to reduce complications and maintain patient safety. In its hospital evaluation criteria and PGY/resident physician training guidelines, the Ministry of Health and Welfare has explicitly stipulated that mastery of the clinical skills associated with the insertion, monitoring and care of a central venous catheter is mandatory for trainees at all levels. It is hoped that before trainees perform procedures on actual patients, they undergo hands-on training with simulators to enhance their skills in ultrasound-assisted CVC placements. This invasive procedure, often performed in race-against-time situations for critically ill patients, represents a significant challenge for both the patient and the administering physician. Beyond just successful placement, the overarching goal in modern medical education and training is to ensure that physicians can swiftly and effectively execute catheter placement, thereby minimizing complications.

# Ultrasound-Guided Central Venous Catheter Placement

Traditionally, ultrasound-guided CVC placement can be broken down into the following six steps [15]:

1. Identify the anatomy of the insertion site and localize the vein.

(1) Identify the right internal jugular vein, common carotid artery, thyroid gland, trachea, and sternocleidomastoid muscle.

(2) Check for anatomic variations.

(3) Use short axis (transverse, in-plane approach) and long axis (longitudinal, out-of-plane approach) view.

(4) Perform this step before prepping and draping of the puncture site.

2. Confirm patency of the vein.

(1) Use compression ultrasound to exclude venous thrombosis.

(2) Use color Doppler imaging and Doppler flow measurements to confirm the patency of the vein and to quantify blood flow.

3. Use real-time US guidance for puncture of the vein.

(1) Use an aseptic approach.

(2) Use a short axis/out-of-plane (A) or a long axis/in-plane (B) approach.

(3) Constantly try to identify and locate the tip of the needle during the needle approach to the vein and puncture of the vein.

4. Confirm the needle position in the vein.

(1) Confirm that the needle tip is centrally placed in the vein before approaching the guide.

Wire

5. Confirm the wire position in the vein.

(1) Confirm the correct position of the guide wire in a short axis (A) and a long axis (B) view

6. Confirm the catheter position in the vein.

(1) Confirm the correct position of the central venous catheter in the vein in a short axis (A) and long axis (B) view.

Although the aforementioned research has shown that using ultrasound guidance for catheter placement can significantly increase the success rate and safety, a significant challenge remains. To obtain the information presented on the ultrasound monitor, the operator's gaze and attention must repeatedly switch to and from the ultrasound monitor during the main surgical task. This switching, where the operator's gaze moves between the ultrasound screen and the patient's puncture site on the neck, while both hands are engaged in different tasks (one hand holding the probe and the other the puncture needle), results in what is medically termed "task interruption."

For instance, taking the right hand as the dominant hand, we can break down the movement as shown in Figure 2. The act of checking the ultrasound screen's information becomes an external interruption in the main surgical task. An "interruption" is defined as switching between two separate tasks, and after completing the second or third task, returning to continue the original task [10]. Research shows that when the human brain encounters an interruption, we have the following reactions: while we are engaged in the first task, and an external stimulus (a second task) presents, we produce an "internal interruption", called an "interruption lag." This internal interruption sends a message to our brain, prompting us to decide whether to switch tasks or not, as illustrated in Figure 3.

If we interrupt the first task to move to the second, after completing the second task, our brain sends a new message, directing us to revert to the content and unfinished steps of the first task. This results in a second lag, called the "resumption lag" [16]. This process not only increases the overall task operation time but may also unduly increase the brain's cognitive load due to the frequent switching of attention. This is particularly challenging for trainees who are not yet familiar with the anatomical structures and task content. This heightened cognitive load can lead to medical errors or the inability to complete the task altogether. Research also indicates that frequent external interruptions can increase the cognitive load on medical personnel [17]. This, in turn, has severe implications in the medical field, as task interruptions or failures can lead to medical errors, potentially causing harm to patients [18-20].


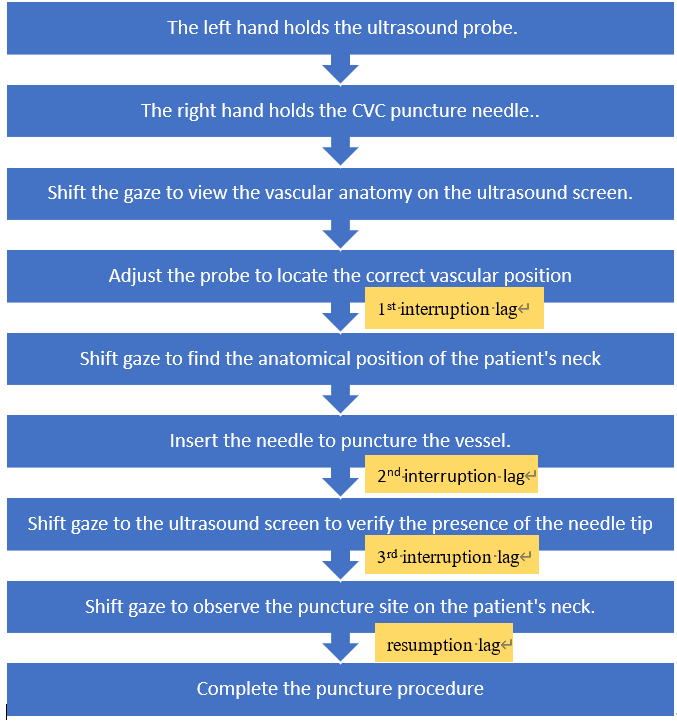


Figure2. Flowchart of CVC insertion procedure.


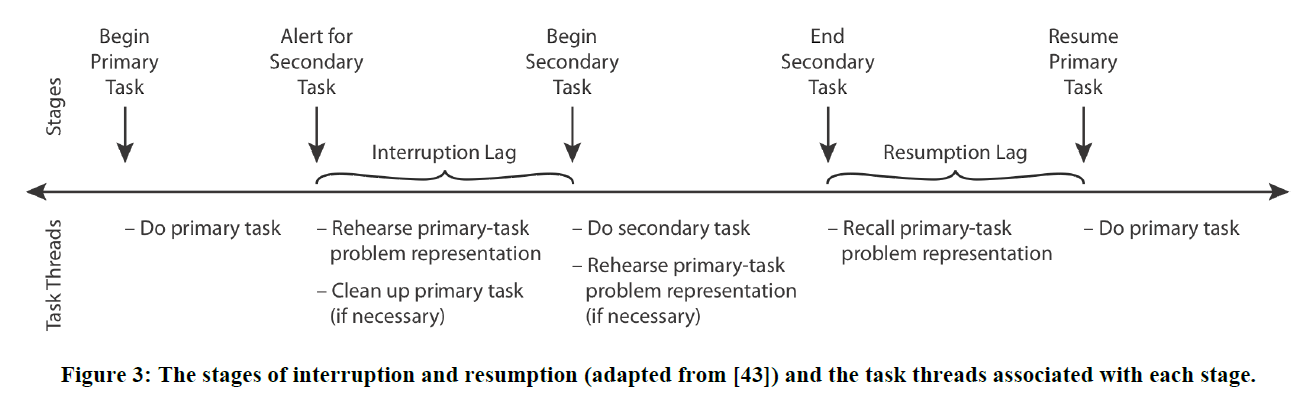


Figure 3. The stages of interruption and resumption (adapted from [43]) and the task threads associated with each stage [21]

# Cognitive Load Theory (CLT)

The Cognitive Load Theory (CLT) was proposed by Australian scholar John Sweller in 1988. He postulated that human memory can be divided into three types: Sensory memory, working memory (or short-term memory), and long-term memory. When stimuli from external sources, which could be visual, auditory, or from other senses, are received, they enter the brain's working memory for processing. Once these pieces of information are integrated and assimilated, some of them become schemas and are stored indefinitely in long-term memory. Subsequently, when new information is encountered, the brain can search these long-term memories to retrieve relevant memories and integrate them. Long-term memory is generally believed to be limitless and can continuously expand [22, 23].

American cognitive psychologist George A. Miller presented a study in 1956, where he posited that, in contrast to the limitless use of long-term memory, working memory can only process a limited amount of information chunks (approximately 7±2 items) at a time [24]. Overloading this capacity can lead to cognitive saturation, thereby limiting learning and recall capabilities.

Dr. Sweller further elaborated three types of cognitive loads that can impact working memory: intrinsic load, extraneous load and germane load [25]. Intrinsic load is related to the inherent difficulty of a task, for example, recalling the dosage of a drug (low load) versus calculating a patient's required dosage based on their kidney function (high load). Extraneous load refers to the difficulty in acquiring information within a task, such as comparing a patient who provides clear medical history (low load) to a bedridden patient with impaired language function (high load), or interruptions from nurses frequently entering and leaving a consultation room (high load). Yet another example is explaining a procedure while simultaneously providing visual imagery and vocal narration (low load). Germane load, a more complex concept, refers to the construction of methods and processes in the brain to solve problems, which can be applied when encountering similar situations in the future [26-28].

The collective impacts of intrinsic, extraneous and germane loads can exhaust the working memory's capacity, limiting its ability to manage multiple tasks simultaneously. When the working memory is occupied by these cognitive loads, it can lead to interference between tasks. This interference might result in an inability to process multiple pieces of information simultaneously, leading to unsuccessful task transitions, eventual task errors, or the inability to complete tasks, as illustrated in Figures 4 and 5 [16, 26, 27].


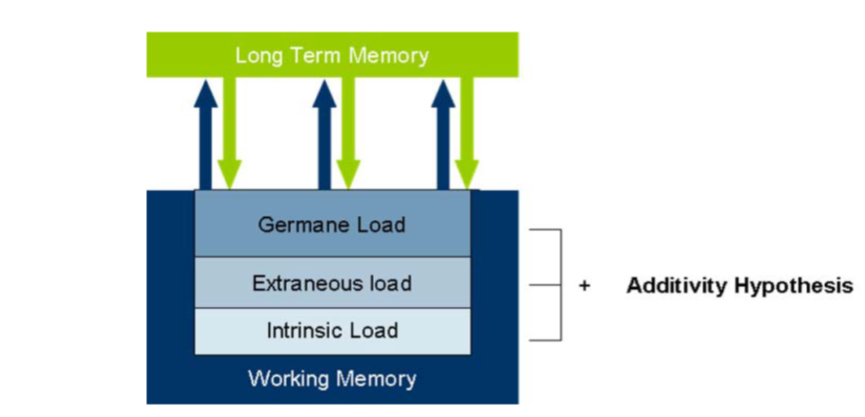


Figure 4. Cognitive Load Theory and its Additivity Hypothesis [20].


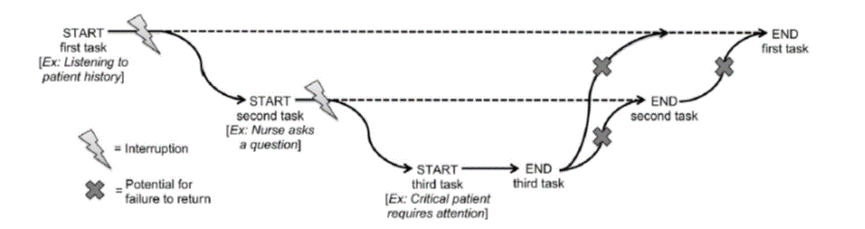


Figure 5. Model of distractions from the task in the ED [26].

Each shift in gaze produces an interruption lag. Every task-switching event may involve a different time of previous task interruption, ranging from milliseconds to several seconds or even minutes. When the operator returns their gaze to the original location, it generates intrinsic and extraneous loads. Specifically:

- Intrinsic load: This involves recalling where they left off in a sequence and the correct order of the steps.
- Extraneous load: The operator has to identify the images on the screen and the anatomical positions in the neck.

This includes identifying at least five structures:

1. Right internal jugular vein
2. Common carotid artery
3. Thyroid gland
4. Trachea
5. Sternocleidomastoid muscle

Adding their relative positions means at least six visual images need to be remembered concurrently. Additionally, as the operator rotates the probe between in-plane and out-of-plane approaches, they are presented with at least twelve different images in a short span of time. The cognitive load of processing these images can overwhelm the working memory, which typically can handle only about 5-7 items in visual perception at once. When working memory is saturated by these cognitive loads, especially with the many intrinsic and extraneous loads generated between task transitions (like the display screen, ultrasound probe, and patient’s neck anatomy), the brain struggles to process all the information simultaneously. This is further compounded by the presence of three or more interruption lags.

According to research by scholars Iqbal and Bailey, there are three factors influencing interruptions: level, task difficulty, and information carry-over. Level refers to the size and complexity of the task. Task difficulty is divided into six levels. Levels three and four are classified as moderately difficult, requiring certain memory processing. Levels five and six not only require memory processing but also entail procedural generation and calculation comprehension. Information carry-over refers to the cognitive effort needed when transitioning between tasks (Table 1) [21]. The interruptions encountered during an ultrasound-guided CVC insertion, regardless of level, task difficulty, and information carry-over, are considered demanding. This is due to the vast amount of information integration and the significant cognitive effort required during task switching. Research has already proven that ultrasound-guided CVC insertion can reduce erroneous punctures (like arterial punctures), increase success rates, and offer real-time monitoring advantages like reduced pneumothorax or hemothorax. However, while these procedures aim to enhance patient safety, it would be regrettable if their effectiveness were diminished due to cognitive over-load and potentially unnecessary interruptions experienced by the operator.


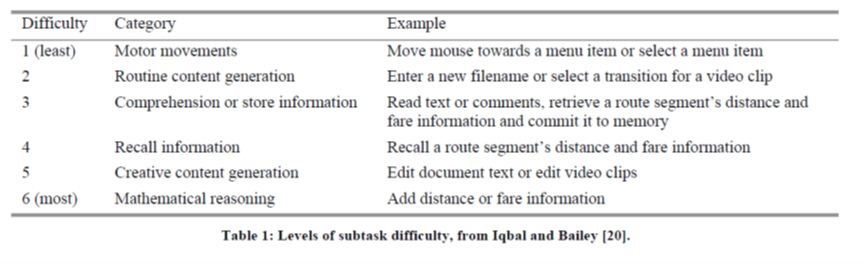


Table 1. Levels of subtask difficulty, from Iqbal and Bailey

# Previous Work Justifying this Study

In certain fields and specialties, external disruptions remain inevitable. Hence, current research focuses on how to reduce the impact of these disruptions through the application of technology, environmental modifications, and through educational training. Thanks to technological advancements, the use of AR (Augmented Reality) smart glasses in medical procedures is growing. The U.S. Food and Drug Administration (FDA) has approved the medical application of Microsoft's HoloLens for pre-operative planning. For instance, a clinical trial in Cleveland, USA, is currently utilizing HoloLens to guide surgeons in tumor surgeries. The University of California, San Francisco (UCSF), has adopted the HTC VIVE VR system to train medical students in surgical procedures using VR patient simulations. Moreover, the team behind Microsoft's HoloLens is collaborating with Philips to develop the Azurion imaging guidance platform based on MR technology. This combines the patient's actual anatomical structures with models generated by computer applications of X-rays and ultrasound images, guiding surgeons during operations.

With the aid of smart glasses, the focal point of surgical viewing can shift from distant to close, moving directly in front of the surgeon. This allows surgeons to perform operations more precisely and engage in simulation training. By shifting the field of view, surgeons no longer need to move their gaze back and forth between patient images (like CT scans, ultrasounds, MRIs) and the surgical site. This can significantly reduce interruptions in medical procedures, thereby decreasing the cognitive load on the surgeon and increasing the chances of success.

Using AR smart glasses, such as Foresee-X Smart Surgical Glasses, can help reduce external disruptions for medical professionals learning ultrasound-guided central venous catheter placement. Foresee-X, illustrated in Figure 6, is a potent augmented reality medical head-mounted binocular display. It can be used during surgeries to display images from ultrasound devices, endoscopes, C-arms, or CT and MRI scans. Through signal capture and image line transmission, images are instantaneously displayed in the Foresee-X smart medical glasses using augmented reality technology. This allows surgeons to focus on the surgical site without needing to shift their gaze to external display monitors, such as traditional PACS systems. Immediate image transmission to the smart glasses allows the upper half of the glasses to display the ultrasound (or CT, MRI) image, while the lower half remains transparent, allowing surgeons a clear view of the surgical site. This intuitive display not only enhances surgical efficiency but also streamlines the entire surgical process. Foresee-X is currently being used in pelvic trauma surgeries, spinal surgery navigation, and other medical treatments and has obtained medical certifications in multiple countries [29].

# Objectives and Hypothesis

Given the aforementioned theories, we see the commonly performed central venous catheterization in intensive care as a case-in-point example to examine the impact of technological assistance (AR smart glasses) on the operator's cognitive load, task interruptions, and final outcome. We will implement a prospective project, using AR smart glasses in ultrasound-guided central venous catheter placement training sessions. By documenting various parameters throughout the procedure, our research will aim to explore the feasibility of this novel technological application in various ultrasound-guided invasive procedure assessments. We are keen to investigate whether technology assistance can reduce the cognitive load on operators, shorten their execution time, and enhance the success rate. After receiving validation through this study, there might be potential in the future to broadly apply AR smart glasses in ultrasound-guided procedures such as thyroid aspirations, breast biopsies, liver punctures, kidney punctures, and any other invasive treatments or assessments requiring ultrasound guidance.


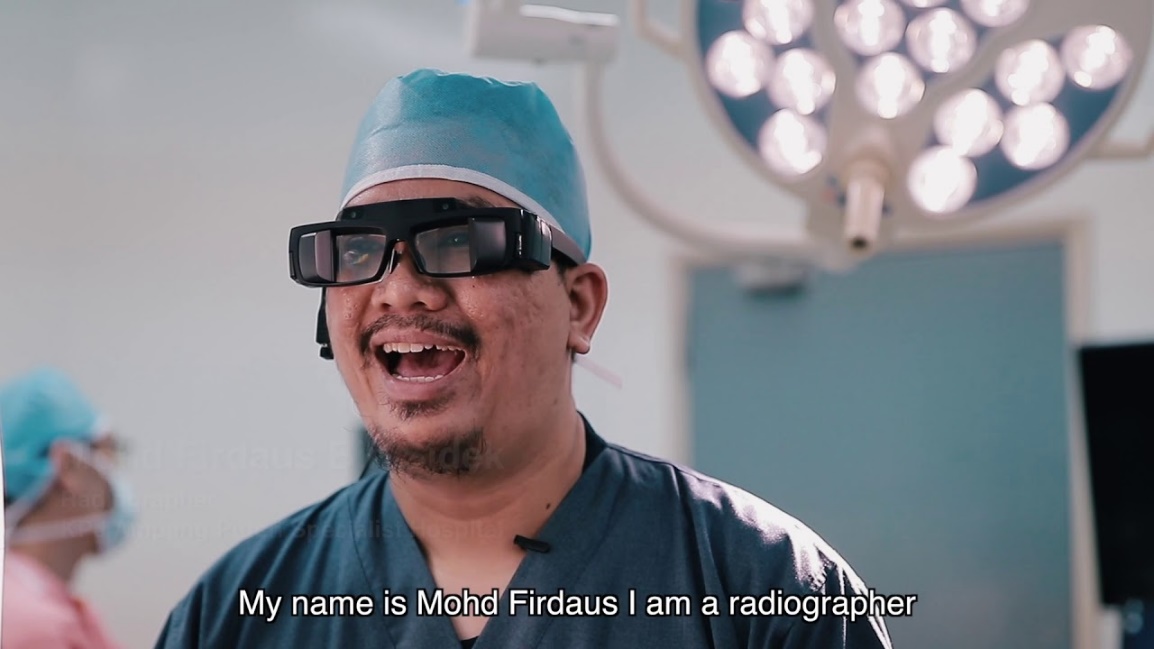


Figure 6. Foresee-X, Smart Surgical Glasses.

# Method

This study will be a prospective randomized crossover trial that uses various devices to investigate whether AR technology can reduce task-switching for healthcare professionals during the execution of critical procedures. The aim is to reduce cognitive load on the operator by minimizing interruptions, thereby increasing the success rate, shortening the execution time, and reducing complications. This will enhance patient safety and improve the quality of medical care.

# 2.1 Existing Central Venous Catheter Training Courses at Chang Gung Hospital

Since August 2020, both Keelung Chang Gung Hospital and Linkou Chang Gung Hospital’s educational departments offer regular courses for the hospital’s resident doctors (from departments including Internal Medicine, Surgery, Obstetrics, Pediatrics, Emergency Medicine, Neurology, and Anesthesia), PGYs, and UGYs. These courses occur monthly, lasting for four hours each session. They are divided into two groups, with each group of 5-6 trainees undergoing two hours of training. Approximately 10-12 trainees are trained each month.

# 2.2 Recruitment Criteria

One to two weeks after the monthly training, trainees from the latest cohort who have completed the course will be recruited for our study on a voluntary basis. Additionally, any physician who has undergone relevant training (ultrasound-guided CVC insertion) and is willing to participate will be included.

# 2.3 Recruitment Methods

Posters will be placed in the Clinical Skills Center (Simulation Medical Education Center). Those who attend the regular courses will receive detailed information. E-mails will be sent to participants of the regular courses inviting them to join the research program, and announcements will be made on educational community websites.

# 2.4 Recruitment Numbers and Sample Size

A power calculation cannot be executed for this research due to the absence of extant data on the application of AR-US in CVC placement. This deficiency precludes the computation of the standard deviation for the primary outcome measure.

- Anticipated enrollment in Linkou CGMH (L): 50.
- Anticipated enrollment in Keelung CGMH (K): 50.

# 2.5 Trial Procedures, Randomization

After a verbal explanation from the principal investigator and the project staff, participants will be enrolled in the study. Each participant in the district will be assigned a number, ranging from L 1-50 for Linkou and K 1-50 for Keelung.

Prior to entering the trial, participants will fill out a questionnaire, then undergo a pre-test. After the pre-test, they will proceed to the main trial.

For each trial, participants will be randomly assigned (by coin-toss) into one of two groups. Group A will first perform the ultrasound-guided CVC insertion using AR smart glasses to display the US images (AR-US). After completing the task, they will rest for 10 minutes and then perform the ultrasound-guided CVC insertion using a standard US monitor (S-US) without AR glasses. Group B will first perform the ultrasound-guided CVC insertion using S-US, without AR glasses. They will then rest for 10 minutes, and then perform the insertion using AR-US. Each participant must complete the following steps during the CVC insertion activity:

1. Identify anatomy of the insertion site and localization of the vein.
2. Confirm patency of the vein. (Target vessel: right internal jugular vein)
3. Use real-time US guidance for puncture of the vein.
4. Confirm needle position in the vein.
5. Confirm wire position in the vein.
6. Confirm catheter position in the vein.

# 2.6 Trial Flowchart

1. Trainees enrolled.
2. Completion of trainees’ basic information questionnaire.
3. Written pre-test, ten multiple-choice questions.

S-US guided CVC

10 Mins rest

S-US guided CVC

NASA Task Load Index

NASA Task Load Index

AR-US guided CVC

10 Mins rest

AR-US guided CVC

NASA Task Load Index

NASA Task Load Index

Analysis

Randomly assigned.

**Before study entry**

**Phase 1**

**Phase 2**

Figure 7. Study flowchart. * The parameters collected include the following: First pass rate, number of attempts, venous access time (seconds), catheterization time (seconds), artery puncture times, total time, success/failure rates, lag time (ultrasound to venous access time), and the number and duration of operator head turns. .

# Preferred Trial Timetable

Preferred Start Date: 1st August 2021

Expected Date of Completion: 31st July 2022

# 2.8 Task Simulator

CAE Gen II Ultrasound Central Line Training Model, Opaque Insert. (Figure 8)


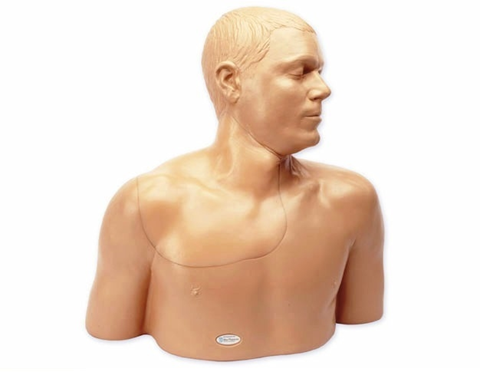


Figure 8. CAE Gen II Ultrasound Central Line Training Model

# 2.9 Smart Glasses

Foresee-X Smart Surgical Glasses produced by Taiwan Main Orthopedic Biotechnology Company (Figure 9)


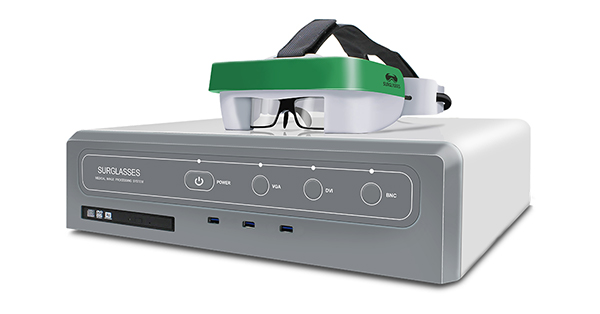


Figure 9. Foresee-X Smart Surgical Glasses.

# Data Collection

3.1 Plans for Assessment and Collection of Outcomes

# 3.1.1 Characteristics of participants

Collect participant information (questionnaire): Data collected includes name, gender, position/rank, dates of course attendance, past experience with CVC insertion, and past experience with ultrasound-guided CVC insertion.

# 3.1.2 Pre-test

Preliminary assessment of participants: Ten questions, derived from past National Physician Examination questions, will be used primarily to test the participants' knowledge about central venous catheter placement but also as a reference for scoring intrinsic load (IL).

# 3.1.3 Primary Outcomes: Performance of CVC Placement

Parameters to be assessed during the CVC placement process include: first pass rate, number of attempts, venous access time (in seconds), catheterization time (in seconds), number of artery punctures, total time, success/failure rate, lag time (from ultrasound to venous access), and the number and duration of operator head turns. There are two methods for collecting these parameters. The first is through continuous recording to collect data on the time spent by the operator, number of punctures, head movement durations, etc. The other method uses the WOMBAT - Work Observation Method by Activity Timing software, developed by Macquarie University.

# 3.1.4 Secondary outcomes: Cognitive load Evaluation

Cognitive Load Questionnaire Collection: This includes intrinsic load (IL), extraneous load (EL), and germane load (GL) experienced during the experiment. Three scales are used for assessment: the Paas cognitive load scale, the NASA Task Load Index, and the Rating Scale Mental Effort (RSME). All three scales are employed to evaluate the participant's IL, EL and GL."

# 3.2 Data Management

All files of the participants, including personal data and recorded outcomes from the trials, will be password-protected and stored in a secure location within the principal investigator's academic institution. These files will only be accessible to the principal investigator.

# 3.3 Confidentiality and Fairness

All data related to the participants will be maintained with the utmost confidentiality. The research team will refrain from disclosing any participant-specific information to unauthorized parties. Participation in the study will not have any bearing on a participant's performance during their training. The research team firmly commits to not divulging the participant's performance to mentors or any individuals involved in their performance assessment. Additionally, the research team will abstain from providing any evaluation related to the participant's training at the hospital.

# 3.4 Dissemination

The study's findings are slated for publication in a peer-reviewed journal, aiming to enlighten both participants and medical professionals. Given that the video recordings of implant procedures have been anonymized for analysis, participants will not have access to individual outcomes. Furthermore, no participant will be specifically named or identified in any publications stemming from this research.

# Data Analysis

The research data will be processed using IBM® SPSS® Statistics v26. The data will be presented as mean values and standard deviations (for continuous variables) or counts and percentages (for categorical variables). The primary analysis will employ a 2 x 2 ANOVA (within: intervention method, between: sequence) to statistically compare the continuous variables – specifically: success rate, placement time, and cognitive load. If the sphericity assumption is violated, the Greenhouse-Geisser estimate will be used to adjust the degrees of freedom. All effects will be considered significant at p < .05.

# References

1. Wheelock, A., et al., The Impact of Operating Room Distractions on Stress, Workload, and Teamwork. Ann Surg, 2015. 261(6): p. 1079-84.

2. Sartang, A.G., et al. Evaluation of Rating Scale Mental Effort (RSME) effectiveness for mental workload assessment in nurses. 2016.

3. Paas, F., et al., Cognitive Load Measurement as a Means to Advance Cognitive Load Theory. Educational Psychologist, 2003. 38(1): p. 63-71.

4. Steuer, J., Defining Virtual Reality: Dimensions Determining Telepresence. Journal of Communication, 2006. 42(4): p. 73-93.

5. Azuma, R.T., A survey of augmented reality. Presence: Teleoper. Virtual Environ., 1997. 6(4): p. 355–385.

6. Tang, K.S., et al., Augmented reality in medical education: a systematic review. Can Med Educ J, 2020. 11(1): p. e81-e96.

7. Jain, N., et al., An augmented reality tool for learning spatial anatomy on mobile devices. Clin Anat, 2017. 30(6): p. 736-741.

8. Kamphuis, C., et al., Augmented reality in medical education? Perspect Med Educ, 2014. 3(4): p. 300-11.

9. Chaballout, B., et al., Feasibility of Augmented Reality in Clinical Simulations: Using Google Glass With Manikins. JMIR Med Educ, 2016. 2(1): p. e2.

10. Troianos, C.A., et al., Special articles: guidelines for performing ultrasound guided vascular cannulation: recommendations of the American Society of Echocardiography and the Society Of Cardiovascular Anesthesiologists. Anesth Analg, 2012. 114(1): p. 46-72.

11. Grebenik, C.R., et al., NICE guidelines for central venous catheterization in children. Is the evidence base sufficient? Br J Anaesth, 2004. 92(6): p. 827-30.

12. Lau, C.S. and R.S. Chamberlain, Ultrasound-guided central venous catheter placement increases success rates in pediatric patients: a meta-analysis. Pediatr Res, 2016. 80(2): p. 178-84.

13. Sidoti, A., et al., Ultrasound- versus landmark-guided subclavian vein catheterization: a prospective observational study from a tertiary referral hospital. Sci Rep, 2019. 9(1): p. 12248.

14. Wu, S.Y., et al., Real-time two-dimensional ultrasound guidance for central venous cannulation: a meta-analysis. Anesthesiology, 2013. 118(2): p. 361-75.

15. Saugel, B., T.W.L. Scheeren, and J.L. Teboul, Ultrasound-guided central venous catheter placement: a structured review and recommendations for clinical practice. Crit Care, 2017. 21(1): p. 225.

16. Park, B. Testing the additivity hypothesis of cognitive load theory. 2010.

17. Chisholm, C.D., et al., Emergency department workplace interruptions: are emergency physicians "interrupt-driven" and "multitasking"? Acad Emerg Med, 2000. 7(11): p. 1239-43.

18. Peabody, C.R. and D. Mandavia, Deep Needle Procedures: Improving Safety With Ultrasound Visualization. J Patient Saf, 2017. 13(2): p. 103-108.

19. Institute of Medicine Committee on Quality of Health Care in, A., in To Err is Human: Building a Safer Health System, L.T. Kohn, J.M. Corrigan, and M.S. Donaldson, Editors. 2000, National Academies Press (US)

Copyright 2000 by the National Academy of Sciences. All rights reserved.: Washington (DC).

20. Grundgeiger, T. and P. Sanderson, Interruptions in healthcare: theoretical views. Int J Med Inform, 2009. 78(5): p. 293-307.

21. Salvucci, D.D., N.A. Taatgen, and J.P. Borst, Toward a unified theory of the multitasking continuum: from concurrent performance to task switching, interruption, and resumption, in Proceedings of the SIGCHI Conference on Human Factors in Computing Systems. 2009, Association for Computing Machinery: Boston, MA, USA. p. 1819–1828.

22. van Merriënboer, J.J. and J. Sweller, Cognitive load theory in health professional education: design principles and strategies. Med Educ, 2010. 44(1): p. 85-93.

23. Barrouillet, P., et al., An empirical test of the independence between declarative and procedural working memory in Oberauer's (2009) theory. Psychon Bull Rev, 2015. 22(4): p. 1035-40.

24. Miller, G.A., The magical number seven, plus or minus two: some limits on our capacity for processing information. 1956. Psychol Rev, 1994. 101(2): p. 343-52.

25. Sweller, J., Cognitive load during problem solving: Effects on learning. Cognitive Science, 1988. 12(2): p. 257-285.

26. Skaugset, L.M., et al., Can You Multitask? Evidence and Limitations of Task Switching and Multitasking in Emergency Medicine. Ann Emerg Med, 2016. 68(2): p. 189-95.

27. Polit, D.F. and C.T. Beck, The content validity index: are you sure you know what's being reported? Critique and recommendations. Res Nurs Health, 2006. 29(5): p. 489-97.

28. Fraser, K.L., P. Ayres, and J. Sweller, Cognitive Load Theory for the Design of Medical Simulations. Simul Healthc, 2015. 10(5): p. 295-307.

29. Wu, J.R., et al., Real-time advanced spinal surgery via visible patient model and augmented reality system. Comput Methods Programs Biomed, 2014. 113(3): p. 869-81.
